# Group B *Streptococcus* detection in pregnant women: comparison of qPCR assay, culture, and the Xpert GBS rapid test

**DOI:** 10.1186/s12884-019-2681-0

**Published:** 2019-12-30

**Authors:** Laura L. Vieira, Amanda V. Perez, Monique M. Machado, Michele L. Kayser, Daniela V. Vettori, Ana Paula Alegretti, Charles F. Ferreira, Janete Vettorazzi, Edimárlei G. Valério

**Affiliations:** 1 Department of Gynaecology and Obstetrics Postgraduation Program in Health Sciences: Gynaecology and Obstetrics (PPGGO), School of Medicine (FAMED), Clinical Hospital of Porto Alegre (HCPA). Federal University of Rio Grande do Sul (UFRGS), Porto Alegre, RS Brazil; 2 Serviço de Obstetrícia e Ginecologia, Hospital de Clínicas de Porto Alegre, Rua Ramiro Barcelos, 2350/1124, Santa Cecília, Porto Alegre, RS CEP: 90035-903 Brazil; 3 Department of Molecular Biology, Clinical Hospital of Porto Alegre (HCPA), Porto Alegre, RS Brazil

**Keywords:** Group B *Streptococcus*, *Streptococcus agalactiae*, Xpert GBS, Real-time polymerase chain reaction, Antenatal care

## Abstract

**Background:**

Group B *Streptococcus* (GBS) is one of the most important causative agents of neonatal sepsis. As administration of prophylactic antibiotics during labor can prevent GBS infection, routine screening for this bacterium in prenatal care before the onset of labor is recommended. However, many women present in labor without having undergone such testing during antenatal care, and the turnaround time of detection methods is insufficient for results to be obtained before delivery.

**Methods:**

Vaginal and anorectal specimens were collected from 270 pregnant women. Each sample was tested by Xpert GBS, qPCR, and culture for GBS detection.

**Results:**

The overall prevalence of maternal GBS colonization was 30.7% according to Xpert GBS, 51.1% according to qPCR, and 14.3% according to cultures. Considering the qPCR method as the reference, the Xpert GBS had a sensitivity of 53% and specificity of 93%. Positive Xpert GBS results were correlated to marital status (married or cohabitating) and with prematurity as a cause of neonatal hospitalization. Positive cultures were related with ischemic–hypoxic encephalopathy requiring therapeutic hypothermia.

**Conclusions:**

Combined enrichment/qPCR and the Xpert GBS rapid test found a high prevalence of GBS colonization. The Xpert GBS technique gives faster results and could be useful for evaluating mothers who present without antenatal GBS screening results and are at risk of preterm labor, thus allowing institution of prophylactic antibiotic therapy.

## Background

Group B *Streptococcus* (GBS) is a gram-positive bacterium associated with the colonization of human body’s mucous membranes. GBS is one of the most important causative agents of neonatal sepsis, which can be prevented by administration of prophylactic antibiotics during labor. Women can be transiently, intermittently, or persistently colonized by GBS in their vaginal or anorectal mucosae [1]. A prevention strategy based on bacterial screening and intrapartum antimicrobial prophylaxis in those pregnant women identified as carriers has led to a reduction in the incidence of neonatal diseases attributable to GBS [2]. The U.S. Centers for Disease Control and Prevention (CDC) [3] recommend routine screening for GBS as an integral part of antenatal care. Ideally, this should be done at a gestational age of 35 to 37 weeks, or earlier in women at risk of premature labor.

Routine GBS screening is done by polymerase chain reaction (PCR)-based tests and cultures. A 2011 study that evaluated a combination of enrichment culture and PCR versus conventional cultures at Hospital de Clínicas de Porto Alegre (HCPA), a tertiary care hospital in Southern Brazil [4], found that enrichment culture/PCR had 87% specificity, with a positive predictive value of 59% and a negative predictive value of 100%. Since 2015, enrichment culture with real-time polymerase chain reaction (qPCR) has become the standard method for GBS detection at HCPA [4–6]. However, these methods usually take around 48–72 h to complete, which has prompted the search for a more rapid test, especially to support urgent decision-making regarding administration of antibiotic prophylaxis.

The Xpert® GBS (Cepheid) is a rapid test based on qPCR technology whereby rectal and vaginal swabs are collected and a result is obtained in approximately 50 min. This method is commercially available in Brazil and has demonstrated high sensitivity for GBS detection in other studies [7, 8].

Considering that many women in Brazil present in labor without having undergone GBS screening during antenatal care, and that many women at risk of preterm labor are admitted to maternity units before GBS screening can be performed on an outpatient basis, the turnaround time of conventional GBS detection methods is too slow for results to be obtained before delivery. Within this context, the present study was designed to evaluate the diagnostic accuracy of the Xpert GBS rapid test and compare it with that of combined enrichment/qPCR (currently used for GBS screening at HCPA) and with the conventional vaginal/rectal discharge culture method.

## Methods

This prospective study was carried out between March and September 2017. Pregnant women who presented for medical appointments at the outpatient, antenatal, and labor and delivery units of the HCPA (Porto Alegre Clinic Hospital) Department of Obstetrics and Gynecology were recruited. The study (number: 2016–0560) was approved by the Research Ethics Committee of Gynecology and Obstetrics Research and Postgraduate Group (GPPG-GO) and conducted in accordance with the provisions of the Declaration of Helsinki. All patients provided written informed consent prior to enrollment. In case of participants under 18 years of age, the guardian also signed the consent form. The inclusion criterion was gestational age ≥ 24 weeks, while the exclusion criterion was any use of antibiotics in the 30 days preceding enrollment.

**Fig. 1:**
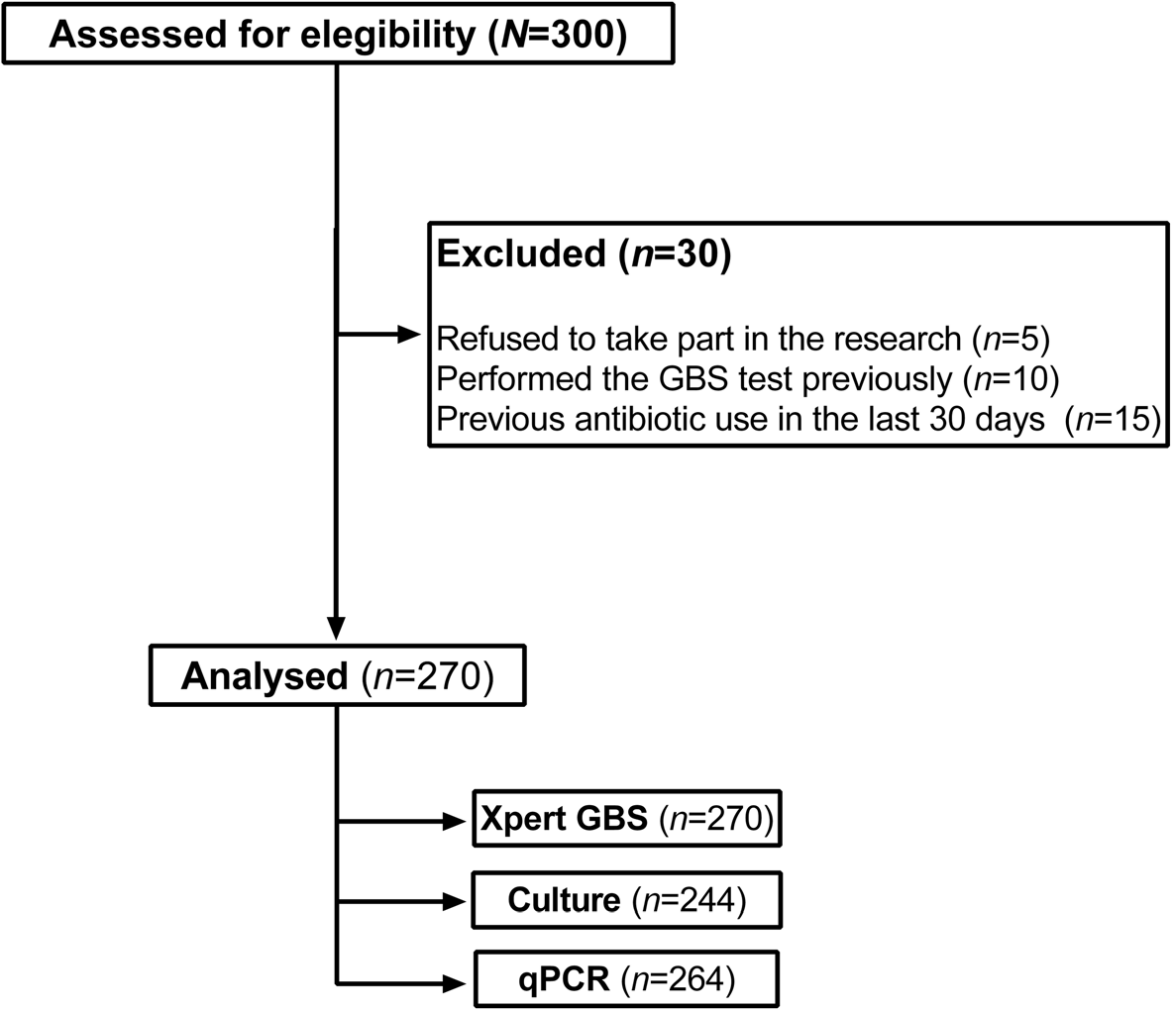
Patiente selection flowchart

Among 300 enrolled women, 30 were excluded: 5 refused to participate, 10 had already undergone GBS screening and received their results at the time of study inclusion, and 15 had used antibiotics in the last 30 days (Fig. 1). Thus, the final sample comprised 270 women.

Three vaginal and rectal swabs (one sample for each screening method – Xpert GBS, qPCR, and culture) – were collected from each patient and immediately stored in Stuart transport medium, according to CDC recommendations. The swabs collected for qPCR were sent to the HCPA microbiology and molecular biology laboratories; the culture swabs were sent to an outside laboratory (Endocrimeta®); and the Xpert GBS samples were analyzed on specific equipment provided by Cepheid. All samples were sent for evaluation within 24 h of collection.

### Xpert® GBS (Cepheid)

The collected swab was transferred to the designated chamber of the Xpert GBS cartridge, which was loaded into a Cepheid GeneXpert device, as recommended by the manufacturer. A trained physician performed all Xpert GBS assays. The result could be negative or positive based on the detection of the target gene sequence adjacent to the GBS *cfb* gene, as defined by the GeneXpert software. Xpert GBS performs automation and integration of sample lysis, amplification and purification of nucleic acids, and detection of the target sequence using qPCR. The total assay runtime was around 50 min.

### GBS culture

The collected swab was inoculated on blood agar plates and incubated at 37 °C for 48 h in a microbiological incubator. After incubation, the plates were inspected for the presence of beta-hemolytic colonies. If there was any suspicion of beta-hemolytic plaque growth after 48 h, plaques were reincubated for another 24 h and inspected again. Beta-hemolytic colonies whose morphology were consistent with GBS were subcultured and CAMP-tested [9]. CAMP-positive colonies were deemed presumably positive for GBS.

### Real-time polymerase chain reaction (qPCR)

Sample preparation and DNA (deoxyribonucleic acid) extraction.

The swabs were incubated for 18 to 24 h into Todd Hewitt selective medium containing gentamicin and nalidixic acid. After centrifugation of the broth, the precipitate was washed with 1X PBS (phosphate-buffered saline) solution (pH = 7.2) and centrifuged again. Then, the precipitate was washed with 1X Tris-EDTA (TE) buffer (10 mM Tris-HCl, 0.1 mM EDTA, pH = 7.5), and DNA extracted by thermal lysis. The thermal lysis protocol was performed using TE solution for 15 min at 100 °C followed by 15 min at − 80 °C to lyse bacterial cell walls. The quality and quantity of DNA extracted from samples were estimated spectrophotometrically in a Nanodrop ND-1000 system (Thermo Fisher Scientific, USA), at 260 nm (A260) and 280 nm (A280) absorbance, with the sample diluted to 5 ng/μL.

### Real-time polymerase chain reaction

For the qPCR, we used the *cfb* gene region that encodes the CAMP factor present in GBS. The primers used were 5′-TTT CAC CAG CTG TAT TAG AAG TA-3′ and 5′-GTT CCC TGA ACA TTA TCT TTG AT-3′. For internal control, a synthetic single-chain DNA (5′-ATC GCT GAT CCG GCC ACA TAT CGC GTT TAT GCG AGG TCG GGT GGG CGG GTC GTT AGT TTC GTT TTG GGC CTA CGT GGC CTT TGT CAC CGA-3′) was used to detect amplification inhibition in all samples using the primers 5′-ATC GCT GAT CCG GCC ACA-3′ and 5′-TCG GTG ACA AAG GCC ACG TA-3′.

The amplification reagents were prepared as follows: Platinum® SYBR® Green (Invitrogen) concentrated mix 6.25 μL, SBG primers 1.25 μL, ROX 1:50 0.25 μL, and DNAse- and RNAse-free water 2.5 μL; 0.5 μL of internal control (IC) solution and 0.75 μL of primers were added to the IC tube.

The extracted DNA solution was added to 10 μL of amplification reagents. Amplification and fluorescent detection were measured by qPCR in a 7500 Real-Time PCR System (Applied Biosystems). The amplification was performed with one cycle at 50 °C for 2 min for DNA polymerase activation, followed by one cycle at 95 °C for 10 min for initial denaturation, than 40 cycles at 95 °C for 15 s and 60 °C for 1 min for amplification, followed by two cycles at 95 °C for 15 s and 60 °C for 15 s for fluorescence detection and melting temperature (Tm) measurement. Samples are considered positive when the amplification curve is detected and the Tm is in the acceptable range (GBS Tm 76–78 °C and IC Tm 82–84 °C). The negative control should not have an amplification curve for the GBS target, while the positive control should be positive for the two targets tested. To ensure high sensitivity in the PCR, the cutoff point was set at a threshold cycle value of C_t_ = 40 [10, 11].

#### Statistical analysis and sample size

Sample size was calculated in WinPEPI Version 11.63, based on the findings of a previous study [4]. Considering a 16% prevalence of GBS positivity with the gold-standard method (culture) and 95% power to detect a 5% difference in prevalence of a positive result, with an estimated 10% attrition rate, the final sample size required was 230 participants.

Regarding the data processing, we used a database double entry, and review were performed using the SPSS, version 18.0. [SPSS Inc. Released 2009. PASW Statistics for Windows, Version 18.0. Chicago: SPSS Inc.]. Symmetric data was expressed as mean and standard error of mean (±SEM), or by median and interquartile range [Percentiles 25th–75th, P25–P75]. The Shapiro-Wilk test was used to determine the normality of data distribution. Categorical variables were described as absolute (n) and relative (n%) frequencies.

Agreement between assays was determined using the kappa and Cronbach’s alpha coefficients. The sensitivity, specificity, negative predictive value, and positive predictive value of the tests were evaluated in accordance with STARD (Standards for Reporting of Diagnostic Accuracy) initiative recommendations.

Spearman’s ρ coefficients were estimated for obstetric characteristics and GBS positivity.

The level of significance was set at 5% for all analysis.

## Results

The maternal characteristics and main neonatal outcomes are listed in Tables 1 and 2. The median [P25–P75] maternal age was 29 [22.0–35.0] years, 39.3% were nulliparous, 72.6% were white, and 82.6% were single or not living with a partner. Women with a gestational age < 35 weeks and any risk of preterm labor represented 48.7% of the sample. Considering fetal characteristics and neonatal outcomes, most were not premature (66.3%) and, among them, were categorized as moderate to late preterm (86.9%). In addition, most newborns had no fetal malformations (96.1%). A rate of 2.5% of neonatal death was observed, with median [P25–P75] Apgar score, 5-min, of 9.0 [9.0–10.0]. Most newborns (62.4%) were not admitted to the Neonatal Intensive Care Unit (NICU).

**Table 1:** Obstetric data of the women included in the study

Variable	Total (***n*** = 270)
Age (years), md [P25–75]	29.0 [22.0–35.0]
Race/ethnicity, n (%)	
White	196 (72.6)
Nonwhite	74 (27.4)
Marital status, n (%)	
Single or not living with a partner	223 (82.6)
Married or cohabitating	47 (17.4)
Educational attainment, n (%)	
Incomplete primary education	64 (23.7)
Completed primary education	48 (17.8)
Incomplete secondary education	38 (14.1)
Completed secondary education	83 (30.7)
Incomplete higher education	24 (8.9)
Completed higher education	13 (4.8)
Gestational age at sampling (weeks), md [P25–75]	35.0 [32.7–36.1]
Gestational age < 35 weeks (days), n (%)	131 (48.7)
Gestational age at birth (weeks), md [P25–75]	38.6 [36.4–39.5]
Parity, n (%)	
Nulliparous	106 (39.3)
Primiparous	72 (26.7)
Multiparous	92 (34.1)
Number of antenatal appointments, md [P25–75]	8.0 [5.0–11.0]
Mode of delivery, n (%)	
Cesarean	134 (49.6)
Vaginal	126 (46.7)
Missing	10 (3.7)
Complications, n (%)	
Yes	33 (12.2)
No	227 (84.1)
Missing	10 (3.7)
Type of complications, n (%)	
Uterine hypotonicity	21 (58.3)
Chorioamnionitis	6 (16.7)
Reintervention	4 (11.1)
ICU admission	2 (5.6)
Postpartum fever	2 (5.6)
Endometritis	1 (2.8)
Sample collection setting – n (%)	
Inpatient	103 (38.1)
Outpatient	167 (61.9)

*n* absolute frequency, *%* relative frequency, *md* median, *P25–75* interquartile range [percentiles 25th–75th], *ICU* intensive care unit

**Table 2:** Fetal characteristics and neonatal outcomes

Variables	Total (***n*** = 282)^a^
Prematurity – n (%)	
Yes	84 (29.8)
No	187 (66.3)
Missing	11 (3.9)
Preterm birth – n (%)	
Extremely preterm (GA ≤28 weeks)	4 (4.8)
Very preterm (GA 28–32 weeks)	7 (8.3)
Moderate to late preterm (GA > 32–37 weeks)	73 (86.9)
Fetal malformations – n (%)	
Yes	11 (3.9)
No	271 (96.1)
Birthweight – n (%)	
Extremely low (≤999 g)	5 (1.8)
Very low (1000–1499 g)	4 (1.4)
Low (1500–2499 g)	67 (23.8)
Adequate (≥2500 g)	195 (69.1)
Missing	11 (3.9)
Neonatal death – n (%)	
Yes	7 (2.5)
No	262 (92.9)
Fetal death	2 (0.7)
Missing	11 (3.9)
Apgar score, 5-min – md [P25–75]	9.0 [9.0–10.0]
Neonatal asphyxia– n (%)	
Yes	17 (6.1)
No	221 (78.9)
Missing	42 (15.0)
NICU admission – n (%)	
Yes	92 (32.6)
No	176 (62.4)
Fetal death	2 (0.7)
Missing	12 (4.3)
Cause of NICU admission – n (%)	
Respiratory distress	49 (53.3)
Jaundice	20 (21.7)
Prematurity	20 (21.7)
Sepsis	19 (20.7)
Fetal malformation	11 (12.0)
Congenital syphilis	10 (10.9)
Hypoglycemia	9 (9.8)
Low birthweight	3 (3.3)
Maternal condition	2 (2.2)
Cyanosis and hypertonia	1 (1.1)
Workup of cutaneous lesions	1 (1.1)
Workup of urinary tract malformation	1 (1.1)
Ischemic–hypoxic encephalopathy	1 (1.1)
Causes of neonatal death – n (%)	
Fetal malformation	5 (71.4)
Extreme prematurity	2 (28.6)

*n* absolute frequency, *%* relative frequency, *md* median; interquartile range [percentiles 25th–75th]; *NICU* Neonatal Intensive Care Unit; ^a^n = 282, including twins

Xpert GBS testing was performed in samples from 270 women; 75 (27.8%) were positive, 169 (62.6%) were negative, 1 (0.4%) was inconclusive, 21 (7.8%) yielded errors, and 4 (1.5%) had no result available, as shown in Table 3. The percentage of errors may be justified by a known and reported problem with a specific batch of cartridges, while four samples were lost due to a power outage (no result).

**Table 3:** Results of antepartum GBS screening by qPCR, Xpert GBS, and culture

Variable	Total (***n*** = 810)	qPCR (n = 270)	Xpert GBS (*n* = 270)	Culture (n = 270)
Status – n (%)				
Positive	245 (30.2)	135 (50.0)	75 (27.8)	35 (13.0)
Negative	507 (62.6)	129 (47.8)	169 (62.6)	209 (77.4)
Inconclusive	1 (0.0)	0 (0.0)	1 (0.4)	0 (0.0)
Error	21 (2.6)	0 (0.0)	21 (7.8)	0 (0.0)
No result	4 (0.5)	0 (0.0)	4 (1.5)	0 (0.0)
Not done	32 (4.0)	6 (2.2)	0 (0.0)	26 (9.6)
Valid results – n	752	264	244	244
Positive	245 (32.6)	135 (51.1)	75 (30.7)	35 (14.3)
Negative	507 (67.4)	129 (48.9)	169 (69.3)	209 (85.7)

*qPCR* real-time polymerase chain reaction, *n* absolute frequency, *%* relative frequency

Only the positive and negative results were included in the analysis. Considering these results alone, the overall prevalence of maternal GBS colonization was 51.1% according to qPCR, 30.7% according to Xpert GBS, and 14.3% according to cultures (Table 3).

We compared the performance of the three tests considering valid results alone (Table 4). A total of 239 women were screened with both the Xpert GBS and qPCR. GBS colonization was detected in 124 (51.9%) with qPCR versus 74 (31.0%) with the Xpert GBS. Considering qPCR as the reference, the Xpert GBS had a sensitivity of 53.2% and a specificity of 93.0%. The positive predictive value was 89.2%, and the negative predictive value was 64.8%. The kappa coefficient between the two techniques indicates moderate agreement (kappa = 0.46), with apparent low sensitivity and high specificity for the rapid test.

**Table 4:** Pairwise comparisons between diagnostic tests used for GBS screening

n (%)		Positive	Negative	Total	^a^***p***-value	Kappa	Cronbach’s α
		qPCR					
Xpert GBS	Positive	66 (53.0)	8 (7.0)	74 (31.0)	≤0.0001	0.456	0.665
	Negative	58 (47.0)	107 (93.0)	165 (69.0)			
	Total	124 (100.0)	115 (100.0)	239 (100.0)			
Culture	Positive	31 (25.4)	3 (2.5)	34 (14.2)	≤0.0001	0.226	0.471
	Negative	91 (74.6)	115 (97.5)	206 (85.8)			
	Total	122 (100.0)	118 (100.0)	240 (100.0)			
		Xpert GBS					
Culture	Positive	21 (31.8)	13 (8.4)	34 (15.5)	≤0.0001	0.271	0.447
	Negative	45 (68.2)	141 (91.6)	186 (84.5)			
	Total	66 (100.0)	154 (100.0)	220 (110.0)			

*n* absolute frequency, *%* relative frequency, *qPCR* real-time polymerase chain reaction, *p* statistical significance

^a^Chi-square test with adjusted residual values

A total of 220 women were screened with both the Xpert GBS and culture methods with valid results. GBS colonization was detected in 66 patients (30.0%) by the Xpert GBS versus 34 (15.5%) with the culture method (Table 4). Considering culture as the gold standard, the Xpert GBS had a sensitivity of 61.8% and a specificity of 75.8%. The positive predictive value was 31.8%, and the negative predictive value was 91.6%. The kappa coefficient between the two techniques indicates fair agreement (kappa = 0.27).

In this study, GBS colonization detected by qPCR was not related to maternal characteristics, such as age, marital status, ethnicity, education attainment, and parity, nor with maternal or neonatal complications, such as chorioamnionitis and sepsis. Positive results with the Xpert GBS rapid test were correlated to marital status (married or cohabitating) and with preterm delivery as a cause of neonatal hospitalization. Finally, ischemic–hypoxic encephalopathy and need for therapeutic hypothermia were related with positive cultures (Table 5).

**Table 5:** Correlations between obstetric characteristics and GBS positivity

Items	Xpert GBS		qPCR		Culture	
	Coefficient	^a^p-value	Coefficient	^a^p-value	Coefficient	^a^p-value
Age	− 0.075	0.242	0.036	0.561	0.047	0.464
Educational level	0.013	0.837	0.039	0.531	0.065	0.314
Single or not living with a partner	**−0.143**	**0.025**	− 0.059	0.342	− 0.040	0.535
Black ethnicity	0.094	0.141	0.054	0.385	0.037	0.562
Parity	−0.002	0.970	0.012	0.844	−0.091	0.154
Maternal complications	−0.087	0.183	−0.012	0.850	0.071	0.279
Chorioamnionitis	0.027	0.680	0.047	0.456	0.008	0.906
Endometritis	−0.044	0.499	0.061	0.334	−0.027	0.676
Intrapartum fever	0.037	0.573	−0.003	0.964	0.091	0.164
Neonatal complications	0.095	0.146	0.009	0.888	−0.002	0.971
NICU admission	−0.099	0.133	0.011	0.867	−0.031	0.641
Cause of NICU admission						
Sepsis	0.035	0.761	−0.053	0.629	0.061	0.587
Prematurity	**0.242**	**0.031**	−0.111	0.310	−0.003	0.976
Respiratory distress	−0.101	0.371	−0.028	0.800	−0.153	0.172
Ischemic–hypoxic encephalopathy	0.195	0.083	0.104	0.343	**0.282**	**0.011**
Hypothermia protocol	0.097	0.139	0.061	0.334	**0.156**	**0.017**

*qPCR* real-time polymerase chain reaction; n, absolute frequency, *%* relative frequency, *NICU* Neonatal Intensive Care Unit, *p* index of statistical significance

^**a**^Spearman correlations. Significance set at 5% for all analyses

*p* value < 0,05 has statistical significance

## Discussion

The overall prevalence of GBS colonization varies depending on the studied population and the test used for screening. This variability may be related to various climatic, biological, sociocultural, geographic, and methodological determinants [12, 13]. In Brazil, prevalence has been reported to range from 9 to 36% [13–15]. In one Brazilian study [14] that compared culture and PCR, only 9.5% of samples were positive for GBS by culture, while 32.6% were positive when using PCR methods. Our study population was restricted to patients of a public hospital that serves as a referral center for high-risk pregnancies, which may explain our finding of a much higher prevalence (51.1%) than is usually reported in the literature, considering the qPCR method. In fact, this is one of the highest prevalence values ever reported among Brazilian women. In a 2011 study [4] conducted at the same hospital as the present investigation but using the conventional PCR agarose gel technique, the prevalence of GBS was 27.0%—much lower than that found in the present study. However, this difference can be justified by the higher sensitivity of qPCR.

In a study [16] carried out among women in the Southeast region of Brazil, the prevalence of GBS by the culture method was around 18%, although only vaginal samples were collected. In an Italian sample of pregnant women [17], about 20% were GBS-positive with the culture method. The prevalence of GBS by culture in the present study (14.3%) was similar to the results of previous studies [4, 14, 16]. The low prevalence of GBS positivity by culture methods as compared to other modalities may be justified by issues of technical execution, as the culture technique does not always follow CDC recommendations [3], or perhaps because this method has a much lower sensitivity than PCR-based methods.

According to the CDC [3], cultures are the gold-standard method for GBS screening in pregnant women at 35–37 weeks of gestational age. The CDC guidelines also cite other laboratory tests for GBS detection, including PCR methods. PCR-based approaches are gaining a promising role in GBS detection, largely due to their higher sensitivity [6, 13, 18, 19]. A European consensus statement noted that failure to treat GBS-positive mothers could lead to serious adverse neonatal outcomes. Thus, it seems reasonable to consider methods with higher sensitivity even if they are associated with more false-positive results.

On comparison of the Xpert GBS to qPCR, we found a high degree of agreement on negative results (93% specificity), but only reasonable agreement on positive results (53% sensitivity). These discordant results can be justified by the higher sensitivity of the combined sample enrichment/qPCR method. As the maternal pathogen load that characterizes actual risk of neonatal infection is unknown, it is unclear whether a real need exists to increase the sensitivity of rapid tests or if their current parameters are sufficient to support clinical decision-making.

Considering culture as the gold standard, the Xpert GBS showed a sensitivity of 62% and a specificity of 76% in this study. According to Gavino [20], the sensitivity and specificity of the Xpert GBS were 95.8 and 64.5% respectively, while those of antenatal cultures were 83.3 and 80.6% respectively. Mueller [21] found a sensitivity of 85.7% and a specificity of 95.6% for the Xpert GBS compared to culture. These divergent results suggest that additional studies are needed to evaluate this method.

In a previous evaluation [17] of the performance of the Xpert GBS rapid test when performed intrapartum, 13.4% of performed tests failed to yield a valid result on the first attempt (7.3% erroneous, 4.4% invalid, and 1.6% yielded no result). Another study [22] reported an invalid result rate of 13.6%, while Mueller [21] reported 13.4% after a 2-h training session on thermocycler operation. In the present study, 90.4% of tests were valid; the remainder were 0.4% inconclusive, 7.8% erroneous, and 1.5% yielded no result. Although some of the errors found in this study may be justified by known problems with a batch of Xpert cartridges, the percentage of invalid tests is consistent with previous reports in the literature.

Positive GBS test results were not related to neonatal sepsis in this study. Considering that GBS infection can be very serious and affects approximately 2% of newborns whose mothers are colonized, a larger sample would almost certainly be needed to demonstrate this association; more probably, the absence of association found in this sample suggests that intrapartum antibiotic prophylaxis is effectively preventing neonatal infection by GBS.

## Conclusion

We found a high prevalence of GBS colonization with PCR-based tests. According to qPCR with prior sample enrichment, 51.1% of samples were positive for GBS. This is among the highest prevalence values ever reported in Brazilian women; additional studies using the same technique are warranted to confirm these findings.

On the other hand, in this study the Xpert GBS test detected a prevalence of GBS colonization among Brazilian women similar to that found in the literature (around 30%). We conclude that the Xpert GBS test may be an option for rapid diagnosis, especially in women at risk for preterm labor and women presenting in labor who did not undergo prenatal GBS testing. This would allow initiation of appropriate antibiotic therapy, as well as reduce hospital costs and prevent development of bacterial resistance to antimicrobials. Furthermore, this would protect asymptomatic newborns whose mothers do not have GBS results available from undergoing unnecessary investigations.

Bacterial cultures for GBS detection, which are currently considered the gold-standard method by the CDC, may be replaced by more sensitive and specific methods, such as different PCR-based techniques.

Several factors interfere with the results of the different tests available for GBS detection. Additional studies are needed to compare the performance of these tests, as well as to compare their findings with clinical outcomes.

## Data Availability

The datasets used and/or analysed during the current study are available from the corresponding author on reasonable request.

## Abbreviations

CDC: Centers for Disease Control and Prevention
DNA: Deoxyribonucleic acid
GBS: Group B *Streptococcus*
HCPA: Hospital de Clínicas de Porto Alegre
IC: Internal control
PBS: Phosphate-buffered saline
PCR: Polymerase chain reaction
qPCR: Real-time polymerase chain reaction
STARD: Standards for reporting of diagnostic accuracy
Tm: Melting temperature
